# Conference on Medical Education

**Published:** 1909-05

**Authors:** 


### BUFFALO MEDICAL JOURNAL.

A Monthly Review of Medicine and Surgery.

*EDITOR:*

WILLIAM WARREN POTTER, M. D.

All communications, whether of a literary or business nature, books for review and exchanges, should be addressed to the editor, 238 DELAWARE AVENUE, BUFFALO, N. Y.

VOL. LXIV. MAY, 1909. No. 10

### Conference on Medical Education

THE Fifth Annual Conference of the Council on Medical Education of the American Medical Association was held at the Auditorium Hotel, Chicago, Monday, April 5, 1909. Two sessions were held, one at 10 a. m., and the other at 2 p. m.

The chairman of the council, Dr. Arthur Dean Bevan, of Chicago, opened the proceedings at the appointed hour with an address in which he dealt with the problems awaiting solution in order to carry medical education standards forward to the proper point, to make them what they should be, to give them a respectability equal to the demand of the times. He reviewed the work of the previous conferences and pointed out the most important improvements that had taken place under the influence of the council. His reference to the work and powers of the state examining boards possessed especial interest. Through the courtesy of Dr. Bevan we are permitted to make an advance excerpt of his remarks on this topic from the address. He said:

THE POWER OF THE STATE BOARDS.

When all is said and done a clear and definite fact remains that the power to control and elevate the standards of medical education lies entirely and alone in the hands of the state boards. It is in the power of the state boards in the majority of the states of the Union this year to so raise the preliminary requirements and to exact fair and complete medical preparation as will secure for such states competent medical practitioners.

Briefly, the present situation of medical education demands for a satisfactory solution (1) the passing of the commercial medical schools and the development of strong medical colleges as the medical departments of universities; (2) endowments both private and state for the proper maintenance of such medical departments.

It has been demonstrated that such endowments could not be secured for the independent medical schools, and further, the experience of the past few years as shown clearly that the modern medical school needs such endowments as it cannot be supported properly by the fees of students; (3) the first and most important public health measure is that each state should demand through its state licensing board complete and thorough medical preparation of those seeking to practise medicine within its boundaries. The desirability and value of the curriculum, with necessary equipment, and the like, from the state board standpoint is to be considered, as is also the question of making the state board examination more of a practical test of the candidate's ability to practise medicine.

The secretary, Dr. N. P. Colwell, next made his report of the work for the year, which included some of the conditions found, of medical colleges, as well as the status of medical education at the present time. Reports were then presented by the chairman of the ten subcommittees on medical curriculum as follows:

Anatomy, including Histology and Embryology, Charles R. Bardeen, Professor of Anatomy, University of Wisconsin, College of Medicine, Madison, Wis; Physiology and Physiologic Chemistry, Elias P. Lyon, Professor of Physiology, St. Louis University, School of Medicine, St. Louis; Pathology and Bacteriology, William T. Councilman, Professor of Pathology, Harvard Medical School, 240 Longwood avenue, Boston; Pharmacology, Toxicology and Therapeutics, Torald Sollmann, Professor of Pharmacology and Materia Medica, Pharmacologic Laboratory, Western Reserve University, Medical Department, Cleveland, O.; Medicine, including Pediatrics and Nervous and Mental Diseases, George Dock, Professor of Medicine, Tulane University of Louisiana, New Orleans; Surgery: General and Special, Charles H. Frazier, Professor of Clinical Surgery, University of Pennsylvania, Department of Medicine, Philadelphia; Obstetrics and Gynecology, Joseph B. DeLee, Professor of Obstetrics, Northwestern University Medical School, Chicago; Diseases of the Eye, Ear, Nose and Throat, George E. de-Schweinitz, Professor of Ophthalmology, University of Pennsylvania, Department of Medicine, Philadelphia; Dermatology and Venereal Diseases, William A. Pusey, Professor of Dermatology and Clinical Dermatology, College of Physicians and Surgeons, Chicago; Hygiene, Medical Jurisprudence and Medical Economics, F. F. Wesbrook, Professor of Pathology and Bacteriology, University of Minnesota, College of Medicine, Minneapolis.

These reports recommended a curriculum which aggregated 4,400 hours. At a meeting of the chairmen held the day previous, the reports had been compared, a few duplications of courses were eliminated, and the total hours for each section reduced as follows:

| *Subject.* | *Hours.* |
|---|---|
| I. Anatomy, including histology and embryology... | 700 |
| II. Physiology and Physiologic Chemistry, including 80 hours of Organic Chemistry .................. | 530 |

| | | |
|---|---|---|
| III. | Pathology and Bacteriology | 500 |
| IV. | Pharmacology, Toxicology and Therapeutics | 240 |
| V. | Medicine, including Pediatrics and Nervous Diseases | 890 |
| VI. | Surgery: General and Special | 650 |
| VII. | Obstetrics, 180 hours, and Gynecology, 60 hours | 240 |
| VIII. | Diseases of the Eye, Ear, Nose and Throat | 140 |
| IX. | Dermatology and Syphilis | 90 |
| X. | Hygiene, Medical Economics and Medical Jurisprudence | 120 |
| | Total | 4,100 |

These figures were voted to represent the maximum requirement of a medical curriculum covering four years of 32 weeks each of actual work. It was the opinion of those present that the curriculum should not be made a hard and fixed requirement with which all colleges should comply. Its chief value was considered to be in its suggestiveness and from the fact that it represented an exhaustive study by over 100 leading medical educators, representing all the subjects included in the curriculum.

In the afternoon the state boards relation to standards and as to the character of the examination were dealt with in the following papers:

Some Results of Higher Standards of Preliminary Education, Richard H. Whitehead, Dean of the Department of Medicine of the University of Virginia, Charlottesville. Discussion.

The Character of the State Medical License Examination, Fleming Carrow, member of the State Board of Registration in Medicine of Michigan, Detroit.

Dr. Whitehead gave the results of higher requirements in his school. While the increase caused a reduction in the number of students it greatly raised the average quality. He added that while the south had some serious educational problems to work out, the Council should not lower its standards on that account. The south needed the standard as a goal to be striven for. In conclusion, Dr. Whitehead said: "The day is not so very far off when there will exist the proper basis for high standards in medical education. Already the leaven of high ideals is working in at least three southern universities. And so, I say, do not worry about the south; it will work out its educational development in due time. In the meanwhile the Council can be of much service by stimulating, encouraging, helping—but not by lowering its standard."

While the subjects presented material of interest the discussions were less spirited than usual, indicating that much of the opposition to advances proposed has disappeared, Dr. Whitehead's final sentence, being an index of that fact. The attendance comprised about one hundred persons.

